# Symbiosis signalling genes negatively regulate root responses to salt stress via the CCaMK-IPD3 module in *Medicago truncatula*

**DOI:** 10.1093/jxb/erag025

**Published:** 2026-01-20

**Authors:** Maria A Contreras Delgado, Velindah Chibomba, Brontë R Thomas, Alistair J Reynolds, Lorelei J Bilham, J Benjamin Miller

**Affiliations:** School of Biological Sciences, University of East Anglia, Norwich Research Park, Norwich NR4 7TJ, UK; School of Biological Sciences, University of East Anglia, Norwich Research Park, Norwich NR4 7TJ, UK; School of Biological Sciences, University of East Anglia, Norwich Research Park, Norwich NR4 7TJ, UK; School of Biological Sciences, University of East Anglia, Norwich Research Park, Norwich NR4 7TJ, UK; School of Biological Sciences, University of East Anglia, Norwich Research Park, Norwich NR4 7TJ, UK; School of Biological Sciences, University of East Anglia, Norwich Research Park, Norwich NR4 7TJ, UK; University of Warwick, UK

**Keywords:** Legume–*Rhizobium* symbiosis, *Medicago truncatula*, nodulation, root, salt stress, signalling crosstalk, symbiosis signalling

## Abstract

Legumes are important sources of dietary protein and are key crops for sustainable agriculture because they fix atmospheric nitrogen via symbiotic interactions with rhizobia bacteria. However, legume plants are particularly sensitive to salt stress, with salinity negatively affecting the development of the root nodule symbiosis. Genes that control salt–symbiosis crosstalk or trade-offs are largely unknown and poorly characterized. To assess the role of symbiosis signalling genes in salt stress, we analysed wild-type and symbiosis signalling mutants of *Medicago truncatula* grown in the presence of NaCl, sorbitol, and/or rhizobia. We assessed root growth, plant biomass, nodule number and gene expression responses in plants exposed to stress. Our findings demonstrate that several symbiosis signalling genes play a previously undescribed role in regulating root responses to salt stress, including a calcium- and calcium/calmodulin-dependent protein kinase (CCaMK) and its interacting partner and downstream transcription factor, IPD3. Our results also show that the identified responses to salt stress are due to sodium toxicity rather than osmotic stress. We conclude that symbiosis signalling genes, including the CCaMK–IPD3 signalling module, may mediate signalling crosstalk between salt stress and symbiosis. These findings open new research avenues to explore how the environment regulates the legume–*Rhizobium* symbiosis.

## Teaser

This work shows that several symbiosis signalling genes play roles in regulating salt-stress responses and that the CCaMK–IPD3 signalling module acts as a key regulator of salt–symbiosis signalling crosstalk.

## Introduction

Salt stress is a major threat to agriculture, with approximately half of all existing irrigated soils worldwide adversely affected by salinization (Pimentel *et al.*, 2004). In Europe, soil salinization affects over 30 million hectares of agricultural land (Daliakopoulos *et al.*, 2016). Salinity is of particular concern for the cultivation of legumes as most legume crops are especially sensitive to salt stress (Tanji and Kielen, 2002). Furthermore, legumes are an important source of dietary protein and are key crops for sustainable agriculture because they fix atmospheric nitrogen via symbiotic interactions with rhizobia bacteria. Salt stress inhibits root nodule formation during the legume–*Rhizobium* symbiosis (de Lorenzo *et al.*, 2007) and also inhibits symbiotic nitrogen fixation, with treatments of 100 mM NaCl decreasing nitrogen fixation rates by 40–90% (depending on the legume species) and resulting in equivalently large yield losses (Nadeem *et al.*, 2019). Establishment of the legume–*Rhizobium* symbiosis is relatively well understood and depends on signalling between the two symbionts (Jhu and Oldroyd, 2023). Rhizobia release diffusible signals, called Nod factors, that trigger initial signalling in the host legume via a well-defined symbiosis signalling pathway. In the model legume *Medicago truncatula*, this symbiosis signalling pathway relies on the perception of Nod factors by the LysM receptor NFP (Bozsoki *et al.*, 2020), the generation of an oscillatory calcium signal (calcium spiking), which is dependent on the cation channel DMI1 and the receptor-like kinase DMI2, and decoding of calcium spiking via a calcium- and calcium/calmodulin-dependent protein kinase (CCaMK) (Singh and Parniske, 2012; Miller *et al.*, 2013). Downstream of calcium spiking and CCaMK, a number of transcription factors are required for nodule formation, including the CCaMK interaction partner IPD3, and the GRAS transcription factors NSP1 and NSP2 (Oldroyd, 2013; Jhu and Oldroyd, 2023). Mature root nodules that fix nitrogen are ultimately formed by coordinating bacterial infection through the epidermis with nodule organogenesis initiated in the cortex (Madsen *et al.*, 2010; Liu *et al.*, 2019). Coordination between the epidermal and cortical programmes is dependent on components of the symbiosis signalling pathway, including the central regulator CCaMK (Rival *et al.*, 2012; Hayashi *et al.*, 2014). Components of the symbiosis signalling pathway are also present in non-legumes, where they are essential for the establishment of symbiotic interactions with arbuscular mycorrhizal (AM) fungi (Gutjahr *et al.*, 2008; Li *et al.*, 2022). Mutation of symbiosis signalling genes results in abolishment or impaired establishment of the symbiosis with rhizobia or AM fungi.

The molecular mechanisms underpinning the regulation of nodulation by salt stress have started to be elucidated, and have recently been reviewed (Chakraborty and Harris, 2022; Singh and Valdés-López, 2023). Expression of the early nodulation marker gene *ENOD11* is regulated by salt in an *NSP2*-dependent manner (Chakraborty *et al.*, 2021), while expression of the nodulation-associated transcription factor *NIN* is also regulated during salt stress in soybean, although by NSP1 and an interacting NAC transcription factor (Wang *et al.*, 2022). Phosphorylation of NSP1 has also been identified to regulate symbiosis signalling and nodule formation during salt stress (He *et al.*, 2021). Together, a clear role for NSP1–NSP2 in regulating salt stress and nodulation signalling has therefore emerged. Work in maize and rice has shown that abiotic stress responses [primarily to water stress and oxidative stress, and mediated by the stress hormone abscisic acid (ABA)] are impaired if the key symbiosis gene *CCaMK* is perturbed (Ma *et al.*, 2012; Shi *et al.*, 2012, 2014; Zhu *et al.*, 2016; Ni *et al.*, 2019; Chen *et al.*, 2021). This work suggests a clear role for CCaMK as a positive regulator of ABA signalling during stress. However, the role of CCaMK during salt stress is less clear. There is contradictory evidence whether *CCaMK* expression is either up-regulated (Pandey *et al.*, 2002) or down-regulated (Yang *et al.*, 2011) during salt stress. Moreover, *Arabidopsis thaliana* plants overexpressing the wheat *CCaMK* gene are more chlorotic when grown in the presence of salt, but seed germination of these overexpression lines is improved under salt stress relative to wild-type seeds (Yang *et al.*, 2011). Yang *et al.* (2011) suggest that CCaMK plays a negative role in stress signalling, but this is at odds with the role of CCaMK during water stress and oxidative stress signalling. In addition, there is no evidence whether other genes of the symbiosis signalling pathway are involved in regulating plant growth during abiotic stress and specifically salt stress. Here, we therefore tested the role played by several symbiosis signalling genes during salt stress and show that many genes of the symbiosis signalling pathway are required for legume roots to respond to salt stress. Overall, our findings provide new data to suggest potential signalling crosstalk and trade-offs between symbiosis formation and salt stress via the symbiosis signalling pathway.

## Materials and methods

### Plant material, growth conditions and phenotyping

Seeds of *M. truncatula* ecotype Jemalong A17 (wild type) or loss-of-function mutants in the same background [*nfp-1*, *dmi1-1*, *dmi2-1*, *ccamk-1* (*dmi3-1*), *ipd3-1*, *nsp1-2*, and *nsp2-2*] were scarified with sandpaper, surface-sterilized in 10% sodium hypochlorite solution, imbibed in sterile water, and plated on 1% deionized water agar. After stratification at 4 °C for a minimum of 3 d, seeds were allowed to germinate overnight at room temperature. For experiments on agar plates, germinated seedlings were transferred to Buffered Nodulation Medium (BNM) (Ehrhardt *et al.*, 1992) supplemented with 0, 100, 150, 175, or 200 mM NaCl, as indicated in the figures. Plates were sealed with micropore tape and the starting position of each root tip was marked on the plate. The plates were placed vertically in a controlled environment room (20 °C light and 21 °C darkness, 16 h photoperiod) for 8 d, after which the root length was determined by measuring the distance to the new position of the root tip. In total, an average of 17 plants were measured per condition across three independent replicates. For experiments in soil, germinated seedlings were transferred to an autoclaved mix of 1:1 (v/v) terragreen:sand and initially watered with sterile liquid BNM containing no NaCl. After 5 d, watering was started with sterile liquid BNM supplemented with 0, 50, or 100 mM NaCl or 0, 100, or 200 mM sorbitol, as indicated in the figures, with watering performed three times per week. Plants were grown in controlled environment rooms (20 °C light and 21 °C darkness, 16 h photoperiod), with root length and plant biomass (fresh weight) measured 21 d and 27 d after the NaCl and sorbitol treatments were started, respectively. In total, an average of 24 or 16 plants were measured per condition for the NaCl or sorbitol treatments, respectively, across three independent replicates. For the nodulation experiments, seedlings were grown in terragreen:sand as described above and inoculated with *Sinorhizobium meliloti* strain 1021 (OD_600_ ∼0.03) at initial planting. Nodule numbers were counted 28 d after the NaCl treatments were started. In total, an average of 24 plants were measured per condition, across three independent replicates.

### Real-time quantitative reverse transcription PCR

Root tissue of 21-day-old plants was flash frozen in liquid nitrogen and stored at −80 °C. Frozen material was ground into a fine powder using a pestle and mortar, and approximately 50 mg of frozen powder was used for RNA extraction using the RNeasy® Plant Mini Kit (Qiagen), following the manufacturer’s instructions. RNA was eluted from the RNeasy spin column using 30 μl of RNase-free water and the quality of the eluted RNA was confirmed by measuring the absorbance at 260 nm and 280 nm using a NanoDrop™ 8000 spectrophotometer (NanoDrop Technologies). Total purified RNA was then treated with Turbo DNAse (Ambion), following the manufacturer’s instructions. To test for the absence of contaminating genomic DNA, a PCR was performed using the GoTaq® G2 Green Master mix (Promega) and primers specific for the housekeeping gene *Elongation Factor 1α* (*EF1α*) (Supplementary Table S1). The PCR conditions were as follows: 30 s at 98 °C, followed by 30 cycles of 10 s at 98 °C, 20 s at 53 °C, and 45 s at 72 °C, followed by 5 min at 72 °C. Genomic DNA from wild-type plants was used as a positive control. The absence of contaminating genomic DNA was confirmed by the lack of a band of 222 bp when the PCR product was analysed by agarose gel electrophoresis. Approximately 1 μg of purified RNA from each sample was also analysed by agarose gel electrophoresis to confirm the presence of the 18S and 28S rRNA double bands. Reverse transcription was performed using the SuperScript™ II Reverse Transcriptase kit (Invitrogen), oligo(dT)17 primer and 1 µg purified RNA, following the manufacturer’s instructions, in a total volume of 50 µl per reaction.

Real-time quantitative reverse transcription PCR (qRT–PCR) was performed using an AriaMx Real-time PCR System G8830A (Agilent Technologies). Reactions were performed in 96-well plates with 5 µl SYBR® Green JumpStart™ Taq ReadyMix™ without MgCl_2_ (Sigma-Aldrich), 2.6 µl 25 mM MgCl_2_, 0.2 µl 10 nM gene-specific forward primer, 0.2 μl 10 nM gene-specific reverse primer, and 2 µl of 1:15 (v/v) cDNA:dH_2_O, with a total volume of 10 μl per well. The PCR cycling conditions were as follows: 4 min at 95 °C, followed by 40 cycles of 30 s at 94 °C, 30 s at 55 °C, 30 s at 72 °C, read fluorescence. Results for each PCR were expressed as threshold cycle (Ct) values. Sequences of the gene-specific primers are listed in Supplementary Table S1. Efficiencies of primer pairs were tested using a dilution series of 10^−1^–10^−5^ ng µl^−1^ of each gene-specific PCR product. It was also confirmed that primers did not amplify control samples without cDNA template. PCRs for each sample were repeated in at least technical triplicate. Ct values for each gene were averaged across the technical replicates for each cDNA sample, and Ct values for the housekeeping genes *EF1α* (Sun *et al.*, 2015) and *ACT* (Ariel *et al.*, 2010) were used as a control. The primer efficiency for each primer pair (confirmed as 90–110%) was used for the calculation of the fold induction of each gene. The fold induction (relative expression) for each biological replicate was calculated for NaCl-treated samples relative to untreated samples, and the final fold induction was calculated by averaging results from all three biological replicates.

## Results

### *CCaMK* negatively regulates root growth during salt stress

**Fig. 1.**
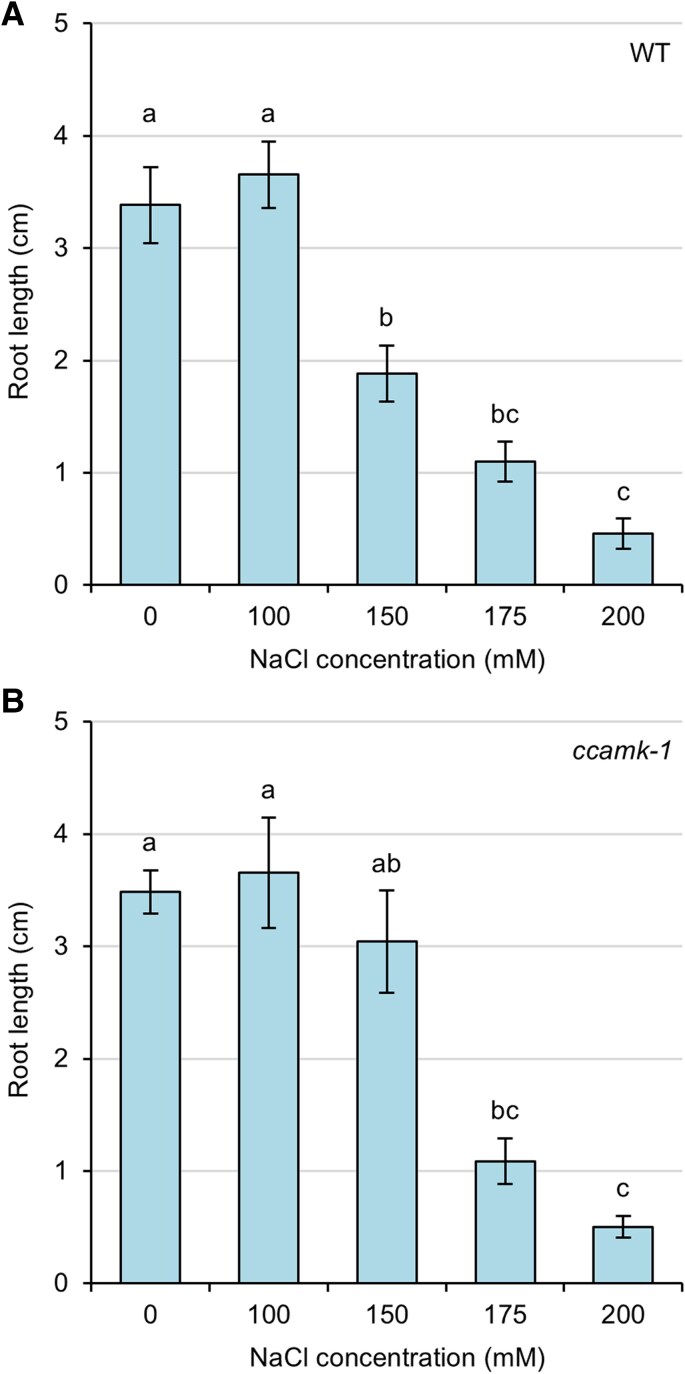
Roots of *ccamk* mutant plants are more tolerant to salt stress than wild-type plants. Root growth was measured in wild-type (A) and *ccamk* mutant (B) plants grown on agar plates and under salt stress for 8 d. Significant differences are indicated by different letters (*P*<0.05; ANOVA with Tukey post-hoc test). Data represent the mean ±SE from three independent replicates. An average of 17 plants were measured per condition.

To determine how salt stress regulates root growth in *M. truncatula*, we grew wild-type and *ccamk* loss-of-function mutant seedlings on agar plates containing different concentrations of NaCl. Both wild-type and *ccamk* mutant plants showed decreased root length in response to increased NaCl concentrations in the medium (Fig. 1A, B). We observed no difference in root length between wild-type and *ccamk* plants grown under control conditions or at 100 mM NaCl (Fig. 1). Unexpectedly, under more severe salt stress, *ccamk* mutant plants had longer roots than wild-type plants (Fig. 1). This difference in root length between wild-type and *ccamk* plants was observed only at 150 mM NaCl and was significant in a pairwise two-tailed *t*-test (*P*=0.036). No difference was observed between wild-type and *ccamk* plants grown under even higher concentrations of NaCl. This response was somewhat unusual and may not have been previously observed because of the relatively narrow NaCl concentration window within which this subtle phenotypic response was detected. Overall, these data show that roots of *ccamk* plants are more tolerant to salt stress than roots of wild-type plants at an intermediate concentration of NaCl, and suggest that *CCaMK* plays a negative role in regulating *M. truncatula* root growth in response to salt stress.

### Several other symbiosis signalling genes also negatively regulate root growth during salt stress

**Fig. 2.**
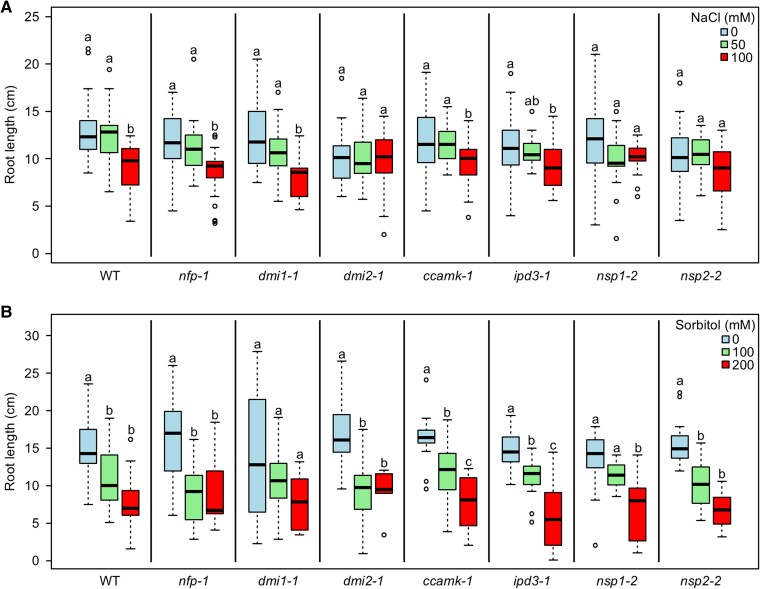
Selected symbiosis signalling mutants are more tolerant to salt stress than wild-type plants. Root length was measured in plants grown in terragreen:sand and under salt stress for 21 d (A) or osmotic stress for 27 d (B). Significant differences are indicated by different letters (*P*<0.05; ANOVA with Tukey post-hoc test). Significance comparisons are within plant genotypes rather than across plant genotypes. Data are from three independent replicates. An average of 24 or 16 plants were measured per condition in (A) and (B), respectively.

To test whether other symbiosis signalling genes or only *CCaMK* is involved in salt-stress responses, we assessed the root lengths of core symbiosis signalling mutants grown under salt stress. These experiments were performed with plants grown in a terragreen:sand mix to determine whether the same root phenotype could be observed under more native conditions than agar plates. For these experiments we also used older plants (rather than seedlings) to determine whether the effect observed in Fig. 1 could also be observed in plants at more advanced growth stages. Interestingly, plants grown in terragreen:sand and subjected to longer treatments with NaCl were more sensitive to salt stress than seedlings grown on agar plates (Fig. 2A). However, under these terragreen:sand growth conditions, wild-type plants still showed a significant decrease in root length upon salt stress, with the roots of plants grown under 100 mM NaCl being 30% shorter than those of control plants (Fig. 2A; Supplementary Fig. S1). No significant decrease in root length was observed in wild-type plants grown under mild salt stress (50 mM NaCl). Of the tested loss-of-function mutants, only *nfp*, *dmi1*, *ccamk*, and *ipd3* plants showed a significant decrease in root length when grown under 100 mM NaCl (Fig. 2A). Importantly, *ccamk* plants showed only an 18% decrease in root length when grown under 100 mM NaCl compared with 0 mM NaCl control conditions. The difference in root length between wild-type and *ccamk* plants at 100 mM NaCl (30% and 18%, respectively) was significant in a pairwise two-tailed *t*-test, thus confirming the phenotype observed on agar plates (Fig. 1) and demonstrating that *ccamk* plants were more tolerant to salt stress. The *nfp* and *dmi1* mutant plants showed decreases in root length of 29% and 35%, respectively, when grown under 100 mM NaCl compared with 0 mM NaCl control conditions (Fig. 2A). Both of these decreases in root length were not significantly different from those seen in wild-type plants, suggesting that *NFP* and *DMI1* are not involved in salt-stress responses. The role of *IPD3* was less clear cut from this root phenotyping analysis, with *ipd3* mutant plants showing a 20% decrease in root length when grown under 100 mM NaCl compared with 0 mM NaCl control conditions. This difference between wild-type and *ipd3* mutants at 100 mM NaCl was just short of the *P*-value cut-off (*P*=0.083). Interestingly, *dmi2*, *nsp1*, and *nsp2* plants showed no significant change in root length when grown under 0, 50, or 100 mM NaCl (Fig. 2A), suggesting these mutants to be less sensitive to salt stress. However, *dmi2* plants were not insensitive to salt stress, as tests on agar plates revealed these mutant plants to respond to higher concentrations of salt (Supplementary Fig. S2). The salt-stress tolerance of *dmi2*, *ccamk*, *ipd3*, *nsp1*, and *nsp2* mutant plants appeared to be root specific, as no equivalent significant difference in total biomass was observed between plants grown under 0 and 100 mM NaCl (Supplementary Figs S1, S3). Overall, our data suggest that the symbiosis signalling genes *DMI2*, *CCaMK*, *IPD3*, *NSP1*, and *NSP2* play a negative role in regulating root growth during salt stress in *M. truncatula*, that is, they are positive regulators of salt-stress signalling.

### Symbiosis signalling genes regulate root growth in response to sodium toxicity rather than the osmotic stress component of salt stress

Since salt stress consists of both ionic stress (sodium toxicity) and osmotic stress components, we grew symbiosis signalling mutants treated with iso-osmolar concentrations of the osmoticum sorbitol to determine if the root-growth phenotype was specifically due to sodium toxicity or osmotic stress. As expected, wild-type plants grown under osmotic stress had shorter roots than those grown under control conditions (Fig. 2B; Supplementary Fig. S4). Significantly shorter roots were observed in wild-type plants grown under both mild and more severe osmotic stress (100 mM and 200 mM sorbitol, respectively) (Fig. 2B). Interestingly, the sorbitol treatments induced a more severe root-shortened phenotype than salt stress at iso-osmolar concentrations (Fig. 2A), with 200 mM sorbitol and 100 mM NaCl causing a decrease in root length of 47% and 30%, respectively, relative to control conditions. The sorbitol treatments caused a significant decrease in root length in all the symbiosis signalling mutants tested except *dmi1* mutant plants (Fig. 2B). We explain this apparent lack of response of *dmi1* mutant plants by the relatively large variation in root length of the *dmi1* mutant plants when grown under 0 mM sorbitol control conditions (Fig. 2B). Importantly, no significant difference in root length was observed between plants of different genotypes grown under 200 mM sorbitol. This demonstrated that the root-growth phenotypes observed with salt stress were not due to the osmotic stress and suggested they were instead due to the sodium toxicity effect of salt stress. The sorbitol treatments also caused a decrease in total biomass (Supplementary Figs S4, S5), but no difference equivalent to that observed for plants treated with salt (Supplementary Fig. S3) was seen between plants of different genotypes grown with sorbitol, again highlighting that the stress response appeared to be due to sodium toxicity rather than osmotic stress.

### Symbiosis signalling genes involved in root responses to salt stress show increased expression

**Fig. 3.**
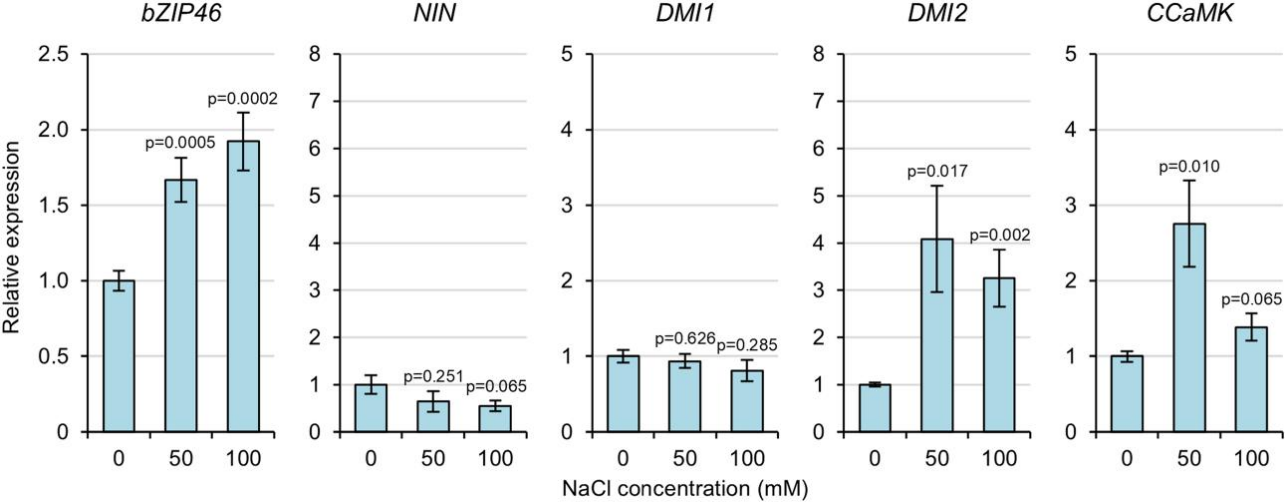
Expression of *DMI2* and *CCaMK*, but not *DMI1*, is increased in roots of plants grown under salt stress. Gene expression was measured by qRT–PCR in roots of plants grown in terragreen:sand and under salt stress for 21 d. Calculated *P*-values in a pairwise two-tailed *t*-test (comparing the specific treatment with 0 mM NaCl control) are indicated. Data represent the mean ±SE from three independent replicates.

To determine whether gene expression correlates with the phenotypes observed, we used qRT–PCR to check the expression of the symbiosis signalling genes in the roots of wild-type plants grown under salt stress (Fig. 2A). We first confirmed the expression of a known stress-induced gene, *bZIP46* (Wang *et al.*, 2015). As expected, *bZIP46* expression showed a significant increase in plants treated with 50 mM and 100 mM NaCl (Fig. 3). To confirm that no symbiotic signalling had occurred in these plants, we also tested the expression of the early nodulation marker gene *NIN* (Marsh *et al.*, 2007) and found no significant changes in expression (Fig. 3). We were thus confident that the plant material had no symbiotic signalling (indeed, no nodules were observed on any roots) and was successfully exhibiting phenotypic (Fig. 2A) and gene expression (Fig. 3) responses specific to salt stress. We therefore next tested the expression of the symbiosis signalling genes and found significant up-regulation of *DMI2* and *CCaMK* expression in roots exposed to salt stress (Fig. 3). Importantly, we did not detect increased expression of *DMI1* (Fig. 3). Increased *IPD3* and *NSP2* expression was also detected during salt stress, although significant variation was observed across replicates (Supplementary Fig. S6). Overall, these findings demonstrate that the expression of symbiosis signalling genes in roots exposed to salt stress correlated with the observed root-growth phenotypes: increased expression was observed for only those symbiosis signalling genes suggested to play a role in salt-stress responses.

### *IPD3* positively regulates the number of root nodules formed during salt stress

**Fig. 4.**
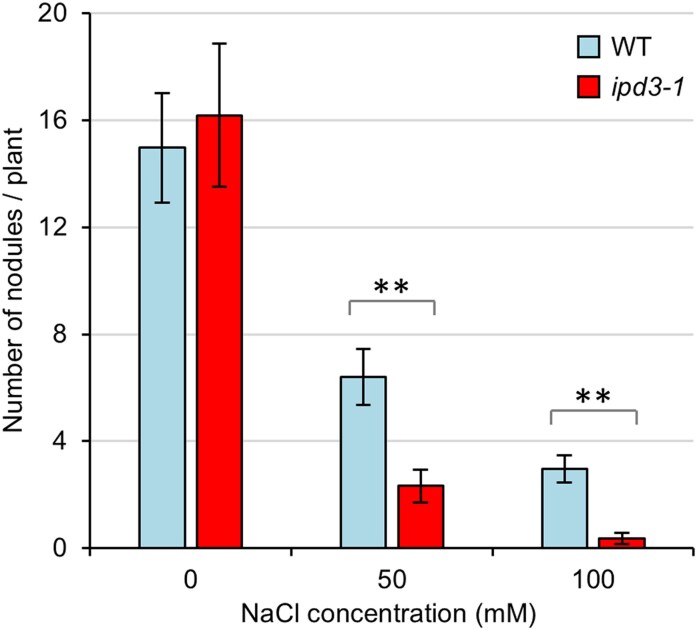
Fewer nodules are formed on the roots of *ipd3* mutant plants than wild-type plants when grown under salt stress. The total number of nodules was counted on roots of plants grown in terragreen:sand and under salt stress for 28 d. Asterisks indicate significance in a pairwise two-tailed *t*-test (**P<0.05). Data represent the mean ±SE from three independent replicates. An average of 24 plants were measured per condition.

To further explore the role of symbiosis signalling components during salt stress, we focused on *IPD3*, since CCaMK–IPD3 act together as a complex, CCaMK is already clearly implicated in abiotic stress signalling, and because *IPD3* is key for controlling bacterial infection via root hairs (Horváth *et al.*, 2011) and nodule organogenesis (Jin *et al.*, 2018). Moreover, *ipd3* plants are one of the few symbiosis signalling mutants still able to form nodules (albeit nodules that are not properly infected by rhizobia). We therefore tested whether *ipd3* plants had altered nodulation in comparison to wild-type plants when grown under salt stress. No difference in the number of nodules formed per plant was detected between wild-type and *ipd3* plants under control (0 mM) conditions, but to our surprise, we found that *ipd3* plants formed significantly fewer nodules than wild-type plants under both 50 mM and 100 mM NaCl (Fig. 4). Although fewer nodules formed on *ipd3* plants grown under salt stress, this was not due to altered plant size, as there was no significant difference in the total biomass of wild-type and *ipd3* plants under these conditions (Supplementary Fig. S7). Overall, these data demonstrate that *IPD3* plays an important role in positively regulating the number of nodules formed during salt stress and suggests that *IPD3* may be a key regulator of salt-stress responses and symbiosis signalling in plant roots.

## Discussion

Our findings demonstrate a new role for several symbiosis signalling genes, including *DMI2*, *CCaMK*, *IPD3*, *NSP1*, and *NSP2*, in regulating root growth during salt stress. Some symbiosis signalling genes, including *NFP*, have previously been implicated in plant–pathogen interactions (Rey *et al.*, 2015; Ried *et al.*, 2019) but, to our knowledge, this is the first report of several genes of the symbiosis signalling pathway being involved in responses to salt stress.

Our results suggest that *NFP* is not involved in regulating root responses to salt stress. The lack of involvement of *NFP* is perhaps not surprising as this gene encodes a receptor for symbiotic Nod factors (Arrighi *et al.*, 2006). Interestingly, *nfp* mutant plants showed a 45% decrease in root length under 100 mM sorbitol treatment (relative to control plants grown under 0 mM sorbitol), while wild-type plants showed a 25% decrease in root length with the equivalent treatments (Fig. 2B). The difference in response between wild-type and *nfp* mutant plants was significant in a pairwise two-tailed *t*-test, suggesting that NFP may also play a role in controlling root length under osmotic stress. A similar response was also observed for *dmi2* mutant plants (43% decrease in root length, which was statistically significantly different from the response observed in wild-type plants). This response is interesting as *DMI2* played a negative role in regulating root growth during salt stress (Fig. 2A). The implications of these results need to be elucidated further, particularly as both genes encode plasma-membrane-located receptors and osmotic stress may interfere with membrane integrity, but it is worth noting that these responses were not observed under the higher concentration (200 mM) sorbitol treatment. It is also noteworthy that no significant difference was observed between wild-type and *nfp* mutant plants under salt stress, suggesting that the NFP response was specific to osmotic stress rather than a response to sodium toxicity. The osmotic-stress treatments used here overall caused a greater degree of stress on the plants than the NaCl treatments, as measured by root length and plant biomass (Fig. 2; Supplementary Figs S3, S5), so it is possible that the osmotic-stress responses observed in the *nfp* and *dmi2* mutant plants were linked to these increased levels of stress. Clearly, the different responses to salt and osmotic stress need further investigation to fully resolve the complexity of the underlying signalling mechanisms and crosstalk between nodulation and salt stress.

Although our data show that *DMI1* has no role in root growth during salt stress, *A. thaliana dmi1* mutants have impaired auxin accumulation and primary root development (Leitao *et al.*, 2019), suggesting that the role of *DMI1* in root growth during salt stress may in fact be more complex. *DMI1* encodes a cation channel essential for nuclear calcium signals associated with symbiosis signalling and root development (Leitao *et al.*, 2019). Likewise, *NUP85*, a component of the nuclear pore complex, is essential for symbiotic calcium signalling (Saito *et al.*, 2007), and has been shown to modulate salt-stress signalling in *A. thaliana* (Zhu *et al.*, 2017). As calcium signals play an important role in salt-stress signalling, it is possible that components of the symbiosis signalling pathway may also contribute to encoding and decoding calcium signals during salt stress. However, when, where, and how such calcium signals occur needs to be addressed with further research, since symbiotic calcium spiking and many of the symbiosis signalling pathway components are associated with the nucleus, while salt-stress-induced calcium signalling primarily occurs at the plasma membrane (Jiang *et al.*, 2019). This difference in spatial location within the cell clearly needs to be resolved. Non-nuclear calcium signals do occur during rhizobial infection of legumes (Miwa *et al.*, 2006; Morieri *et al.*, 2013), but whether these are important in crosstalk during salt stress remains to be seen. Consistent with a possible link is the work of Zahran and Sprent (1986), which shows that salt stress impairs rhizobia colonization and root hair curling during the infection process in *Vicia faba*. It is also worth noting that *CCaMK* and *IPD3* are absent from *A. thaliana*, so, if components of the symbiosis signalling pathway are involved in salt-stress-induced calcium signalling, other calcium-decoder protein components would additionally be required in different (non-symbiotic) plant species.

**Fig. 5.**
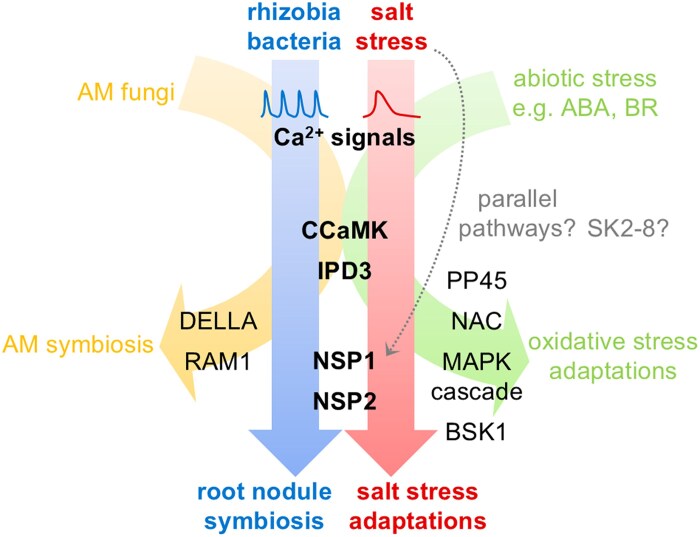
Model for salt stress and symbiosis signalling crosstalk via the CCaMK–IPD3 and NSP1–NSP2 signalling modules. Symbiosis signalling initiated by rhizobia bacteria (blue) or arbuscular mycorrhizal (AM) fungi (yellow) proceeds through calcium spiking and the CCaMK–IPD3 signalling module. The NSP1–NSP2 signalling module is also required for the root nodule symbiosis, but DELLA and RAM1 are involved in the AM symbiosis. Calcium signals play a role in stress signalling pathways, although the signals are different from calcium spiking in the nucleus associated with symbiosis signalling. The CCaMK–IPD3 and NSP1–NSP2 signalling modules are additionally required for salt-stress signalling (red), and CCaMK is central to all pathways, as it has also been implicated in abiotic stress via abscisic acid (ABA) and brassinosteroid (BR) signalling (green). The role of IPD3 in ABA and BR signalling is less clear but, given the role of CCaMK–IPD3 in salt-stress signalling and the role of CCaMK in other abiotic-stress signalling, it would not be unexpected for IPD3 to also play a role, in a similar way to the CCaMK–IPD3–DELLA complex formed during the symbiosis with AM fungi. CCaMK forms additional interactions with other proteins during abiotic stress signalling, including the type 2C protein phosphatase PP45 (Ni *et al.*, 2019), a NAC transcription factor (Zhu *et al.*, 2016; Wang *et al.*, 2022), a MAP kinase kinase (MAPK cascade) (Chen *et al.*, 2021; Yang *et al.*, 2021), and brassinosteroid-signalling kinase 1 (BSK1) (Liu *et al.*, 2021). Parallel signalling pathways (dotted arrow) may also be involved in salt stress [e.g. via the glycogen synthase kinase 3-like kinase SK2-8 (He *et al.*, 2021), which phosphorylates NSP1] and such parallel pathways could be dependent or independent of calcium signalling.

*CCaMK* has previously been described to have roles in abiotic stress signalling, most notably as a positive regulator during ABA signalling (Ma *et al.*, 2012; Shi *et al.*, 2012, 2014; Ni *et al.*, 2019). However, the role of *CCaMK* during salt stress has hitherto proved elusive. Previous work has shown that *CCaMK* expression in pea increases during salt stress (Pandey *et al.*, 2002), and our gene expression data are consistent with this finding (Fig. 3). Moreover, our data show that *CCaMK* negatively regulates root growth during salt stress (Figs 1, 2), demonstrating for the first time a functional role for *CCaMK* specifically during salt stress. The observation that wild-type and *ccamk* mutant plants had slightly different sensitivities to salt stress between the different experiments (Figs 1, 2) may be explained by differences in growth conditions (agar plates vs. terragreen:sand mix) and/or plant growth stage (seedling vs. mature plant). Our finding that *CCaMK* is a positive regulator of salt-stress signalling is consistent with the role of *CCaMK* as a positive regulator of ABA signalling in plants (Ma *et al.*, 2012; Shi *et al.*, 2012, 2014; Ni *et al.*, 2019). CCaMK phosphorylates the transcription factor IPD3/CYCLOPS (Singh *et al.*, 2014), but no role for *IPD3* in abiotic stress has previously been described. However, *IPD3* is a key regulator of bacterial infection (Yano *et al.*, 2008; Horváth *et al.*, 2011) and nodule organogenesis (Singh *et al.*, 2014; Jin *et al.*, 2018). As our data show that *IPD3* regulates both symbiosis signalling and salt-stress responses (Fig. 4), we propose that the CCaMK–IPD3 complex is an important early hub for regulating crosstalk or trade-offs between symbiosis and salt stress (Fig. 5). Indeed, the role of *IPD3* in negatively regulating root growth and positively regulating nodule number suggests that legumes may favour nodulation over root growth during salt stress. Our proposal that CCaMK and IPD3 are together important for regulating salt-stress responses is consistent with the work of Yang *et al.* (2011), who, based on overexpression of the wheat *CCaMK* gene in *A. thaliana* plants, suggest that CCaMK may in fact be a negative regulator of stress signalling. Initially, this work may appear to contradict our findings, but our work suggests that *IPD3* is also required for functional salt-stress signalling and that the result of Yang *et al.* (2011) can therefore be explained by the lack of *IPD3* in *A. thaliana*. More recently, work has shown that CCaMK–IPD3 forms a complex with the DELLA1 protein to regulate signalling via gibberellins during the symbiosis with AM fungi (Pimprikar *et al.*, 2016), and that CCaMK can interact with and phosphorylate a downstream NAC transcription factor (Zhu *et al.*, 2016) and MAP kinase kinase during ABA and stress signalling (Chen *et al.*, 2021; Yang *et al.*, 2021). CCaMK is also regulated during ABA signalling through its interaction with the type 2C protein phosphatase PP45 (Ni *et al.*, 2019). Together, these data support a model of CCaMK–IPD3 as a key signalling module to mediate symbiosis and salt-stress signalling (Fig. 5). We hypothesize that different downstream transcription factors and signalling pathway components may be activated upon different input signals and that this leads to trade-offs and signalling crosstalk between symbiosis and abiotic stress. In further support of this hypothesis, Liu *et al.* (2021) demonstrated that CCaMK is also able to phosphorylate a brassinosteroid-signalling kinase 1 (BSK1) in maize upon drought stress. Interestingly, the authors also showed that phosphorylation of a specific serine residue of CCaMK (Ser-67) positively regulated drought tolerance, raising the further possibility of post-translational modifications such as phosphorylation providing an additional layer in regulating signalling specificity and crosstalk.

Co-evolution of the CCaMK–IPD3 and NSP1–NSP2 protein pairs as functional signalling modules has been proposed (Delaux *et al.*, 2015; Radhakrishnan *et al.*, 2020; Vernié *et al.*, 2025), and involvement of these protein partners in salt-stress responses is consistent with land plant evolution. The evolutionary ancient symbiosis with AM fungi is thought to have allowed plants to colonize the land, but such a dramatic change in environment is likely to have also been associated with abiotic stresses, including salt stress, and the evolution of new responses to adapt to these stresses. In addition to symbiosis, *NSP1* and *NSP2* also regulate strigolactone biosynthesis (Liu *et al.*, 2011; Li *et al.*, 2022), and the NSP1–NSP2 signalling module could perhaps participate in controlling root growth during salt stress via this plant hormone, although crosstalk between strigolactone, auxin, and other hormones may also be possible to regulate root growth. Work in soybean has identified a glycogen synthase kinase 3-like kinase (SK2-8) that phosphorylates NSP1 to inhibit symbiosis signalling and nodule formation during salt stress (He *et al.*, 2021). A soybean NAC transcription factor has also been identified that interacts with NSP1 and regulates *NIN* expression during salt stress (Wang *et al.*, 2022). Whether this signalling through NSP1 is dependent on hormone and/or calcium (and CCaMK–IPD3) signalling, or rather represents a new parallel signalling pathway (Fig. 5), remains unknown; but regardless, the work of He *et al.* (2021), Wang *et al.* (2022), and Chakraborty *et al.* (2021) is consistent with our findings that NSP1–NSP2 plays an important role in regulating salt-stress responses in legumes.

How the CCaMK–IPD3 and NSP1–NSP2 signalling modules are specifically activated during salt-stress signalling, and whether different calcium signals are essential, remain to be elucidated. Recent work has identified AP2/ERF transcription factors involved in salt stress and nodulation in soybean (Zhu *et al.*, 2025), so it will be important to determine how these transcription factors relate to the CCaMK-IPD3 and NSP1–NSP2 signalling modules identified here to further elucidate the mechanisms of salt–symbiosis signalling crosstalk. Moreover, determining how trade-offs between environmental responses and symbiotic nitrogen fixation are controlled, and whether regulation occurs at the level of bacterial infection and/or nodule organogenesis, will be important questions to address in the future.

## Supplementary Material

erag025_Supplementary_Data

## Data Availability

All primary data to support the findings of this study are openly available in Figshare at http://doi.org/10.6084/m9.figshare.31058005 (Contreras Delgado *et al*., 2026).

## Supplementary data

The following supplementary data are available at *JXB* online.

Fig. S1. Representative images of plants grown under salt stress.

Fig. S2. Roots of *dmi2-1* plants are sensitive to salt stress.

Fig. S3. Total biomass of symbiosis signalling mutants grown under salt stress.

Fig. S4. Representative images of plants grown under osmotic stress.

Fig. S5. Total biomass of symbiosis signalling mutants grown under osmotic stress.

Fig. S6. *IPD3* and *NSP2* expression levels in roots of plants grown under salt stress.

Fig. S7. Total biomass of *ipd3-1* and wild-type plants inoculated with rhizobia and grown under salt stress.

Table S1. Primers used in this study.
