# Supplementary material for: Symbiosis signalling genes negatively regulate root responses to salt stress via the CCaMK-IPD3 module in Medicago truncatula
Source: J Exp Bot. 2026 Jan 20;77(8):2506–15. doi: 10.1093/jxb/erag025 (PMC13080356; doi:10.1093/jxb/erag025)
Supplement: erag025_Supplementary_Data [file erag025_supplementary_data.pdf]

SUPPLEMENTARY DATA

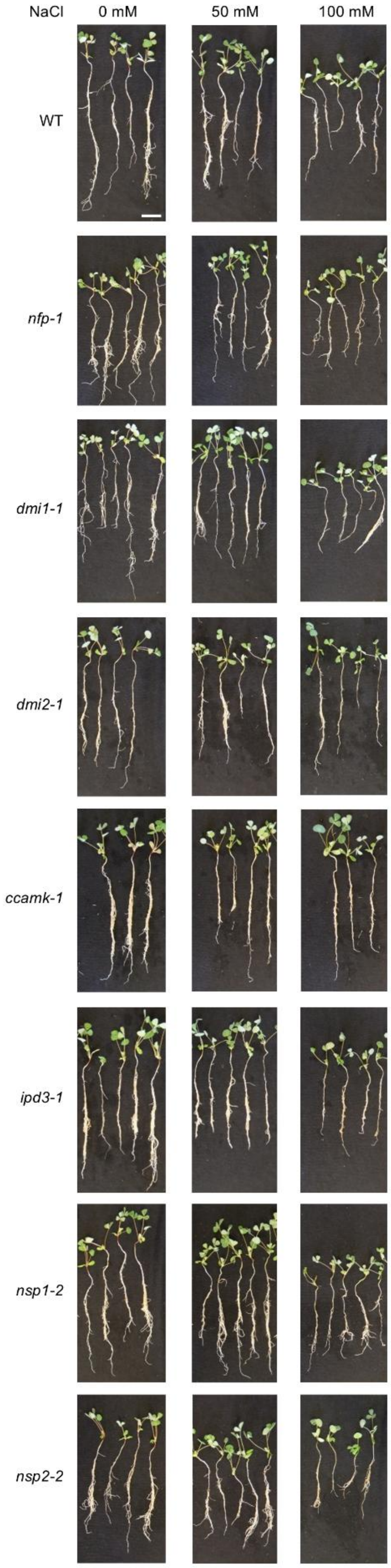

Supplementary Figure S1: Representative images of plants grown under salt stress. Plants were grown in terragreen:sand and under the indicated concentrations of NaCl for 21 days. Scale bar = 2.5 cm.

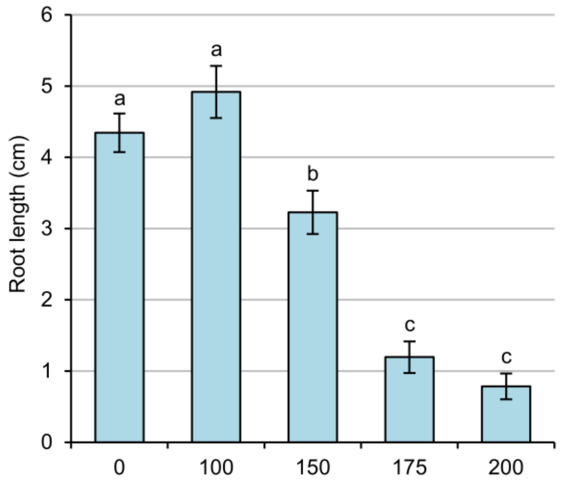

Supplementary Figure S2: Roots of *dmi2-1* plants are sensitive to salt stress. Root growth was measured in plants grown on agar plates and under salt stress for 8 days. Significance in ANOVA with a Tukey post-hoc test is indicated by letters (p<0.05). Data represent mean ± standard error from three independent replicates. An average of 19 plants were measured per condition.

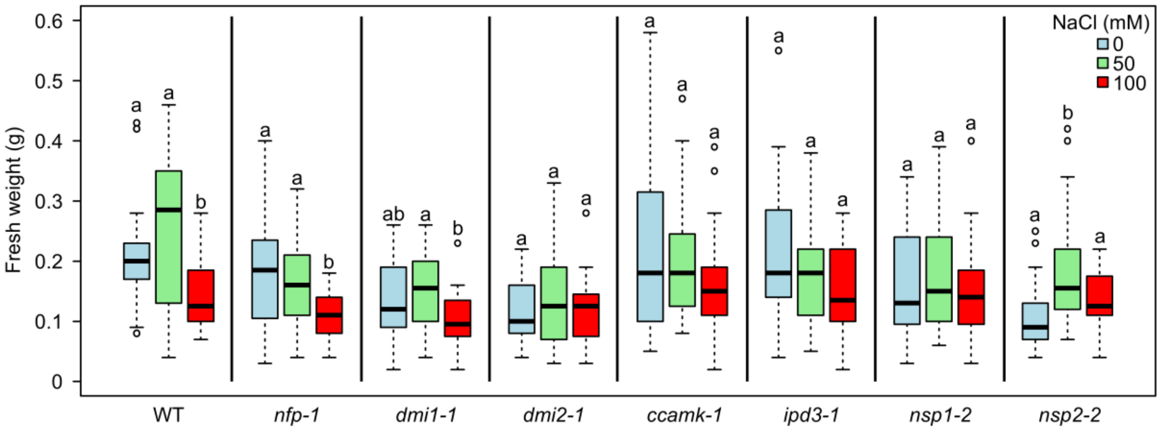

Supplementary Figure S3: Total biomass of symbiosis signalling mutants grown under salt stress. Total biomass was measured in plants grown in terragreen:sand and under salt stress for 21 days. Significance in ANOVA with a Tukey post-hoc test is indicated by letters (p<0.05). Significance comparisons are within plant genotypes rather than across plant genotypes. Data are from three independent replicates. An average of 24 plants were measured per condition.

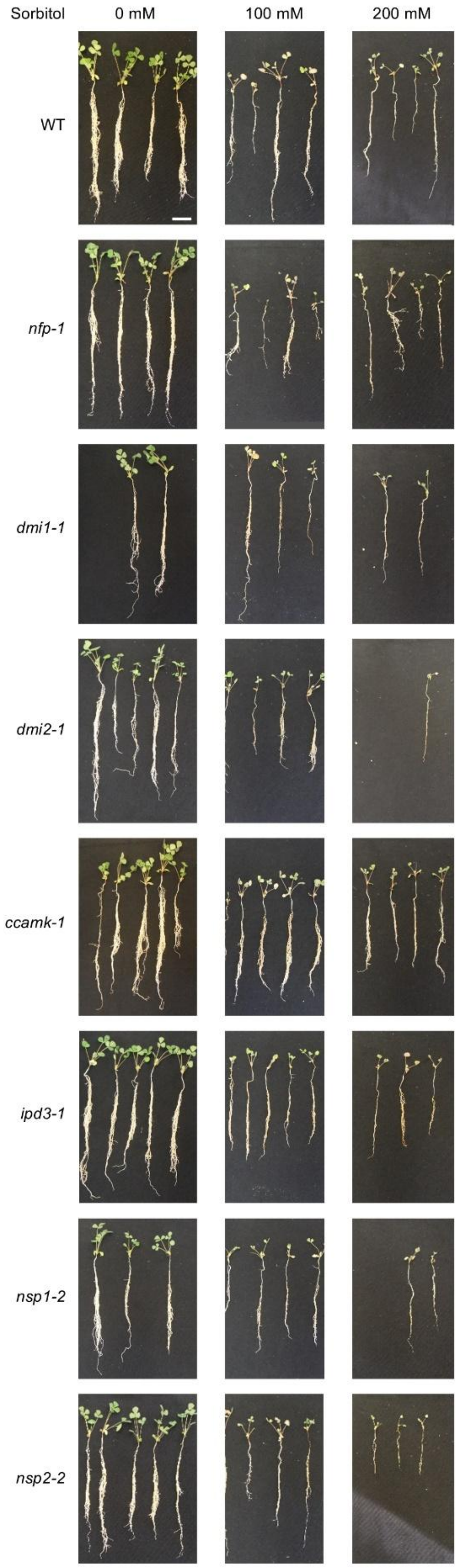

Supplementary Figure S4: Representative images of plants grown under osmotic stress. Plants were grown in terragreen:sand and under the indicated concentrations of sorbitol for 21 days. Scale bar = 2.5 cm.

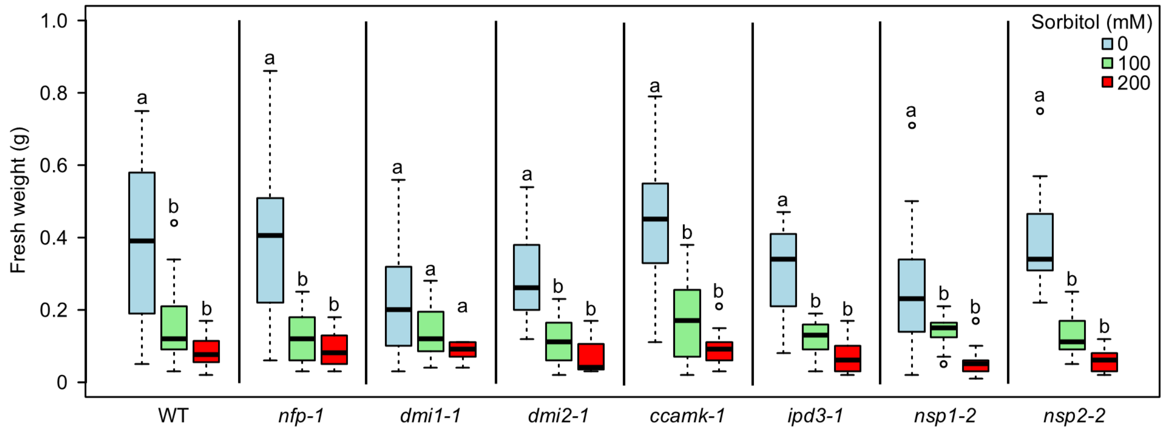

Supplementary Figure S5: Total biomass of symbiosis signalling mutants grown under osmotic stress. Total biomass was measured in plants grown in terragreen:sand and under osmotic stress for 27 days. Significance in ANOVA with a Tukey post-hoc test is indicated by letters ($p<0.05$). Significance comparisons are within plant genotypes rather than across plant genotypes. Data are from three independent replicates. An average of 16 plants were measured per condition.

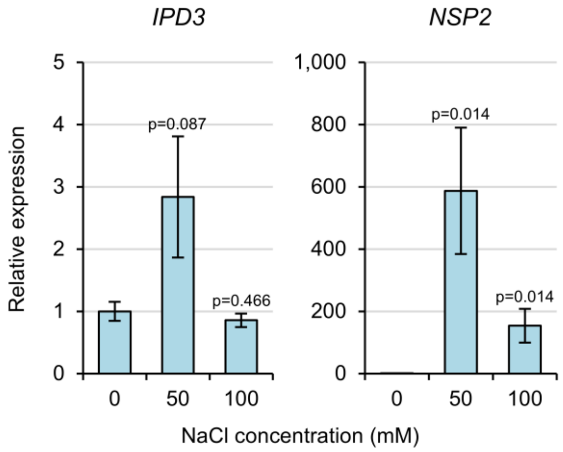

Supplementary Figure S6: *IPD3* and *NSP2* expression levels in roots of plants grown under salt stress. Gene expression was measured by qRT-PCR in roots grown in terragreen:sand and under salt stress for 21 days. Calculated p-values in a pairwise two-tailed t-test (comparing specific treatment to 0 mM NaCl control) are indicated. Data represent mean ± standard error from three independent replicates.

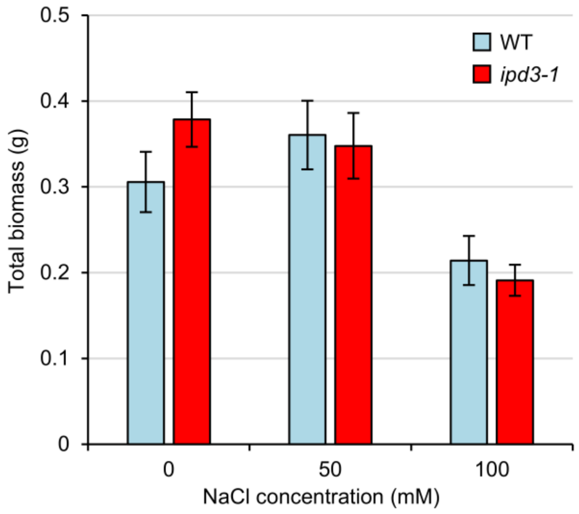

Supplementary Figure S7: Total biomass of *ipd3-1* and wildtype plants inoculated with rhizobia and grown under salt stress. Total biomass was measured in plants grown in terragreen:sand and under the indicated concentrations of salt stress for 28 days. No significant difference was observed between *ipd3* and wildtype plants grown under the same salt stress conditions. Data represent mean ± standard error from three independent replicates. An average of 24 plants were measured per condition.

Supplementary Table S1: Primers used in this study.

Gene	Forward primer	Reverse primer
EF1α	CTTTGCTTGGTGCTGTTTAGATGG	ATTCCAAAGGCGGCTGCATA
ACT	TGGCATCACTCAGTACCTTTCAACAG	ACCCAAAGCATCAAATAATAAGTCAACC
bZIP46	GGAATGGTTGGTTTGGCACC	CCTTTCCTCCCTCGCATACC
NIN	GCAATGTGGGGATTTAGAGATT	GGAAGATTGAGAGGGGAAGCTT
DMI1	TGGTGGTAGCATGGCTGAAG	ATGCCACCCGCACTAATTGA
DMI2	CCTGAGCGATTGGAGTTCGT	CTTGGACCCTCCAGCCAAAA
CCaMK	GGCCTAGTGCTCTTGAGCTT	GCAGCTGCACGAAGTTTACG
IPD3	ACGAATGACAAGAAGCCGGT	TGTGCCCTTTTGGCTTGGTA
